# Naringin Attenuates Autophagic Stress and Neuroinflammation in Kainic Acid-Treated Hippocampus *In Vivo*

**DOI:** 10.1155/2015/354326

**Published:** 2015-06-09

**Authors:** Kyoung Hoon Jeong, Un Ju Jung, Sang Ryong Kim

**Affiliations:** ^1^School of Life Sciences, BK21 Plus KNU Creative BioResearch Group, Kyungpook National University, Daegu 702-701, Republic of Korea; ^2^Institute of Life Science & Biotechnology, Kyungpook National University, Daegu 702-701, Republic of Korea; ^3^Department of Food Science and Nutrition, Kyungpook National University, Daegu 702-701, Republic of Korea; ^4^Brain Science and Engineering Institute, Kyungpook National University, Daegu 700-842, Republic of Korea

## Abstract

Kainic acid (KA) is well known as a chemical compound to study epileptic seizures and neuronal excitotoxicity. KA-induced excitotoxicity causes neuronal death by induction of autophagic stress and microglia-derived neuroinflammation, suggesting that the control of KA-induced effects may be important to inhibit epileptic seizures with neuroprotection. Naringin, a flavonoid in grapefruit and citrus fruits, has anti-inflammatory and antioxidative activities, resulting in neuroprotection in animal models from neurodegenerative diseases such as Parkinson's disease and Alzheimer's disease. In the present study, we examined its beneficial effects involved in antiautophagic stress and antineuroinflammation in the KA-treated hippocampus. Our results showed that naringin treatment delayed the onset of KA-induced seizures and decreased the occurrence of chronic spontaneous recurrent seizures (SRS) in KA-treated mice. Moreover, naringin treatment protected hippocampal CA1 neurons in the KA-treated hippocampus, ameliorated KA-induced autophagic stress, confirmed by the expression of microtubule-associated protein light chain 3 (LC3), and attenuated an increase in tumor necrosis factor-*α* (TNF*α*) in activated microglia. These results suggest that naringin may have beneficial effects of preventing epileptic events and neuronal death through antiautophagic stress and antineuroinflammation in the hippocampus *in vivo*.

## 1. Introduction

Treatment with kainic acid (KA), an analog of excitotoxic glutamate, induces seizures with hippocampal pyramidal neuron death [1]. In addition to excitotoxicity, KA treatment can induce microglial activation, which easily detects the surrounding region of dying neurons [2]. In brain, activated microglia induce proinflammatory cytokines such as interleukin-1 beta and tumor necrosis factor-alpha (TNF*α*), resulting in neuronal cell death involved in excitotoxicity [3–6]. Moreover, KA-induced excitotoxicity induces apoptotic neuronal death, which has been defined as a type II programmed cell death, viainduction of autophagic stress in the striatum [7]. Although it is still controversial whether autophagic stress leads to neuronal death [8], accumulating evidences support that abnormal overactivation of autophagy according to the type and degree of environmental changes or stress stimuli may induce neurodegeneration involved in brain diseases [7, 9–11]. Therefore, these reports suggest that the control of neuroinflammation and autophagic stress induced by excitotoxicity may be important to maintain normal hippocampal system and prevent the induction of epileptic seizures.

Recently, many flavonoids, which can be extracted from natural materials, are highlighted because of their penetration activity of the blood-brain barrier and multiplicity effects to modulate neuronal activities and prevent age-related neurodegeneration [12]. Naringin is a major flavanone glycoside in grapefruits and citrus fruits and has many beneficial properties including anti-inflammatory, anticarcinogenic, lipid-lowering, and antioxidant activities [12–15]. In the previous study, we reported that intraperitoneal injection of naringin could induce neuroprotective effects through anti-inflammatory activities and induction of glia-derived neurotrophic factor as a neurotrophic factor involved in the survival of dopaminergic neurons in the animal model of Parkinson's disease [16]. These results suggest that naringin may be a beneficial natural product to prevent Parkinson's disease, and its effects may be useful in preventing neurotoxicity in another model of brain disease. Recently, there was a report to show the effects of naringin on the onset of seizures induced by KA treatment [17]. However, there was no evidence whether naringin could affect the development of chronic epileptic seizures, and the mechanism involved in naringin-induced antiseizures is still unclear. In the present study, therefore, we investigated additional neuroprotective effects of naringin, including its effects on chronic epileptic seizures, neurotoxicity, autophagic stress, and microglial activation, in a mouse model of KA-treated excitotoxicity.

## 2. Materials and Methods

### 2.1. Animals and Intrahippocampal Injection of KA

Male C57BL/6 mice (8 weeks old, 22-23 g) were obtained from Daehan Biolink (Eumseong, Korea). Mice were housed in a controlled environment and fully provided with food and water. All surgical experiments were performed in accordance with approved animal protocols and guidelines established by the Animal Care Committee of Kyungpook National University (number KNU 2012-37). Anesthetized C57BL/6 mice using chloral hydrate (360 mg/kg, Sigma, St. Louis, MO) were positioned in a stereotaxic frame (David Kopf Instruments, Tujunga, CA). As previously described with modifications [18], each mouse received a unilateral injection of KA (0.2 *μ*g in 4 *μ*L PBS; Sigma) using a 10 *μ*L Hamilton syringe (30 S needle) attached to a syringe pump (KD Scientific, New Hope, PA) into the hippocampus (AP: −2.0 mm; ML: −1.2 mm; DV: −1.5 mm, relative to the bregma). Mice treated with 4 *μ*L PBS (Sigma), which showed no effect on neurotoxicity in the hippocampus, were used as controls. After the injection, the needle was left in place for an additional 5 min before being slowly retracted.

### 2.2. Monitoring of KA-Induced Seizures

After KA treatment, mice were monitored for 6 h to evaluate the onset of seizures. Seizure stage was determined as described previously with some modifications [19]: stage 1: facial movement; stage 2: head nodding, myoclonic twitching, and tremor; stage 3: forelimb clonus with lordotic posture; stage 4: forelimb clonus with reared posture; stage 5: tonic-clonic seizure without postural control. In the present study, mice under stage 3 were determined as positive animals showing the onset of seizure. To examine the effect of naringin on chronic seizures, mice were monitored to evaluate the frequency of spontaneous recurrent seizures (SRS) for 72 h/week (6 h/day, 6 days/week) from 3 weeks after KA or naringin with KA treatment by video recording, as previously described with some modification [20]. Only the following severe seizures (stages 3–5) were considered as mice showing SRS. The frequency of SRS during the monitoring period was quantified.

### 2.3. Naringin Administration

As previously described with some modification [16], mice received a daily intraperitoneal injection of naringin (80 mg/kg per day; Sigma), suspended in 0.25% carboxymethylcellulose (Sigma) which is dissolved in 0.9% saline, starting 1 day before KA injection and continued daily until 6 days after injection. To investigate tumor necrosis factor-alpha (TNF*α*) and microtubule-associated protein light chain 3 isoform B (LC3B) expression, which could indicate neuroinflammation and autophagic stress, respectively, mice received injections of naringin starting 1 day before KA injection and again 1 h prior to KA injection. In addition, to evaluate the effect of naringin on SRS induced by KA treatment, mice received its treatment starting 1 day before KA and daily continued 5 weeks after treatment. Their brains were harvested at the indicated time points for the various analyses.

### 2.4. Immunohistochemical Staining Procedures

Mice tissues for immunostaining were prepared as previously described [16, 21], and the following primary antibodies were used for immunohistochemistry: anti-neuronal nuclei (NeuN, 1 : 200, Millipore, Temecula, CA) and anti-ionized calcium-binding adapter molecule 1 (Iba1, 1 : 2000, Wako Pure Chemical Industries, Japan) overnight at 4°C and then incubated with biotin-conjugated secondary antibodies, followed by an avidin-biotin complex kit (Vector Laboratories, Burlingame, CA). The signal was detected by incubating sections in 0.5 mg/mL 3,3′-diaminobenzidine (DAB, Sigma) in 0.1 M phosphate buffer (PB, Sigma) containing 0.003% H_2_O_2_. Sections were analyzed under a microscope (Axio Imager; Carl Zeiss, Göttingen, Germany). For immunofluorescence labeling, brain sections were incubated overnight with one of the following pairs: anti-NeuN (1 : 200, Millipore) and anti-LC3B (1 : 1000, Cell Signaling, Beverly, MA); anti-Iba1 (1 : 2000, Wako Pure Chemical Industries) and anti-TNF*α* (1 : 500, R&D Systems, Minneapolis, MN). The next day, the sections were incubated with Cy3- (1 : 200, Millipore) and FITC-conjugated IgG (1 : 200, Millipore) and then washed and mounted with Vectashield mounting medium (Vector Laboratories). The stained samples were analyzed under a bright-field microscope (Axio Imager) or a confocal microscopy (LSM700, Carl Zeiss, Germany).

### 2.5. Counting of Hippocampal CA1 Neurons

As previously described with some modification [21], the number of CA1 neurons was counted in the mice hippocampus. Briefly, alternate sections were obtained at 3.3, 3.6, 4.16, and 4.3 mm posterior to the bregma, and two regions from each level (8 regions for each animal) were used to count cells in the CA1 region. The number of neurons within the CA1 layer was counted using a light microscope (Carl Zeiss) at a magnification of 400x and expressed as the number of CA1 neurons per millimeter of linear length. To maintain consistency across animals, a rectangular box (1 × 0.05 mm) was centered over the CA1 cell layer beginning 1.5 mm lateral to the midline, and only neurons with normal visible nuclei were counted. The number of neurons in the ipsilateral hippocampus was expressed quantitatively as a percentage compared to the contralateral control.

### 2.6. Western Blot Analysis

Brain tissues for Western blotting were prepared as previously described [16, 21]. Briefly, the mice hippocampal tissues were homogenized and centrifuged at 4°C for 20 min at 14,000 g. The supernatant was transferred to a fresh tube and the concentration was determined using a BCA kit (Bio-Rad Laboratories, Hercules, CA, USA). Proteins analyzed on gel electrophoresis were transferred to polyvinylidene difluoride membranes (Millipore) using an electrophoretic transfer system (Bio-Rad Laboratories), and then the membranes were incubated overnight at 4°C with specific primary antibodies: anti-*β*-actin (1 : 4000, Cell Signaling), anti-LC3B (1 : 1000, Cell Signaling), and anti-TNF*α* (1 : 1000, R&D system). After washing, the membranes were incubated with secondary antibodies (Amersham Biosciences, Piscataway, NJ), and the blots were finally developed with the ECL Western blotting detection reagents (Amersham Biosciences). For semiquantitative analyses, the density of the immunoblot bands was measured with the Computer Imaging Device and accompanying software (Fuji Film, Tokyo, Japan).

### 2.7. Statistical Analysis

All values are expressed as mean ± standard error of the mean (SEM). Differences between two groups were analyzed by the *t*-test. Multiple comparisons among the groups were performed by one-way analysis of variance (ANOVA) followed by Tukey* post hoc* test. All statistical analyses were performed using the Sigma Stat software (Systat Software, San Leandro, CA).

## 3. Results

### 3.1. Effects of Naringin against KA-Induced Seizures

**Figure 1:**
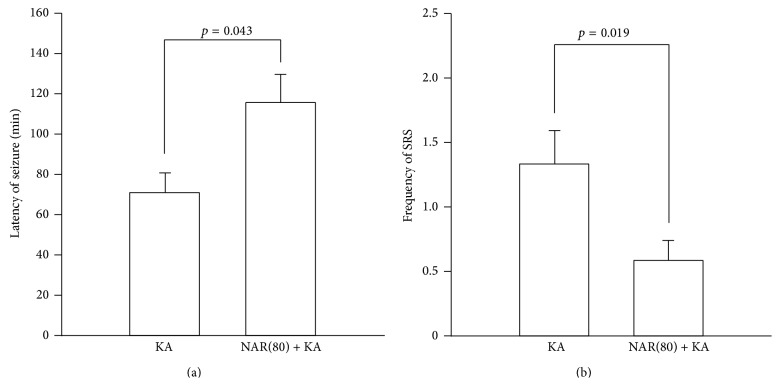
The antiseizure effects of naringin on KA-induced seizures in mice. Mice received twice intraperitoneal injection of naringin (80 mg/kg) at 1 day and 1 h before KA treatment, and then they were treated with intrahippocampal injection of KA to induce seizures. Treatment with naringin significantly increased latency of seizure onset in mice treated with KA compared to KA alone (a). *p* = 0.043, significantly different from KA alone (*t-*test, *n* = 4 for each group). The frequency of SRS evaluated for 2 weeks starting 3 weeks after KA treatment. Mice that received naringin for 5 weeks showed a significant decrease in the frequency of SRS compared to KA alone (b). *p* = 0.019, significantly different from KA alone (*t*-test, *n* = 4 for each group). KA: KA-treated mice; NAR(80)+KA: 80 mg/kg naringin with KA-treated mice.

To clarify the inhibitory effects of naringin against onset of seizures, mice received an intraperitoneal injection of 80 mg/kg naringin [16] starting 1 day before KA injection and again 1 h prior to KA injection. Similar to the previous report [17], our results showed that naringin treatment significantly delayed seizure onset induced by KA treatment compared to KA alone (Figure 1(a), *p* = 0.043). Furthermore, we evaluated whether treatment with naringin could suppress occurrence of chronic spontaneous seizures after KA treatment. Mice received its treatment starting 1 day before KA and daily continued for 5 weeks. Our results showed that naringin treatment significantly decreased the frequency of chronic spontaneous seizures in KA-treated mice compared with KA alone (Figure 1(b), *p* = 0.019), suggesting that naringin might have beneficial properties as an antiepileptic agent.

### 3.2. Neuroprotection by Naringin Treatment in the KA-Treated Hippocampus

**Figure 2:**
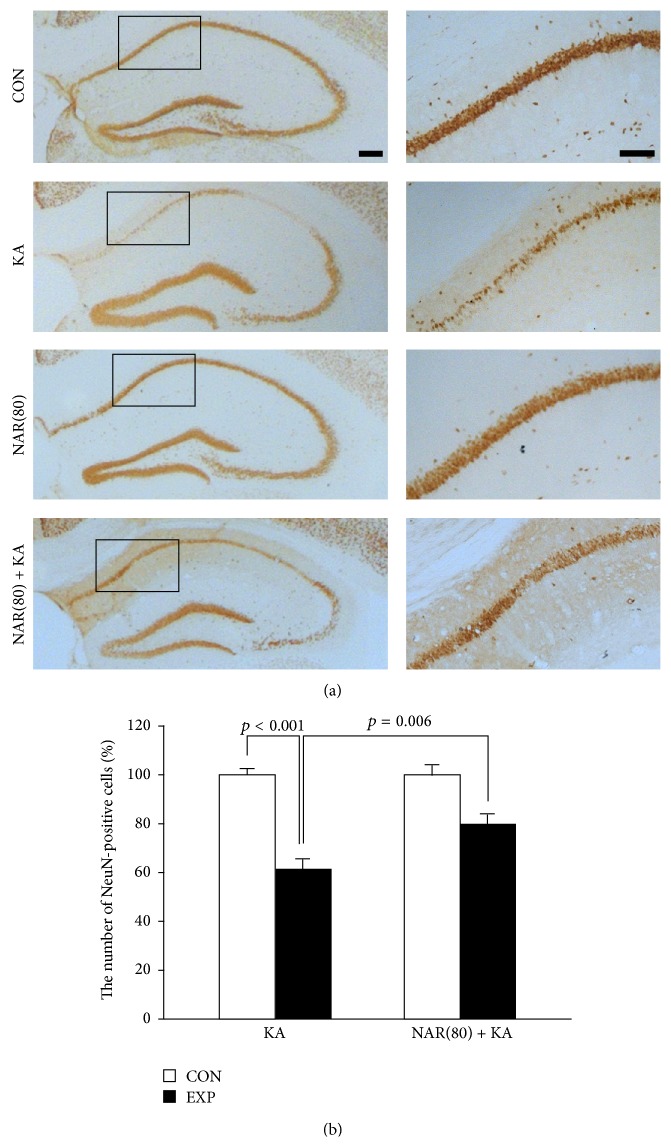
Protective effect of naringin against excitotoxic neuronal death in the KA-treated hippocampus. Naringin was intraperitoneally injected for 7 days, starting 1 day before KA treatment. Representative pictures of immunoperoxidase staining for NeuN are performed at 1 week after KA treatment (a). Both contralateral controls and naringin alone show intact neurons in the hippocampal CA1 region. Seven days after KA treatment, severe neuronal cell death is distinctly found in the hippocampal CA1 area; however, treatment with naringin reduces KA-induced excitotoxic cell death in the CA1 pyramidal neurons. Scale bar: 500 *μ*m and 100 *μ*m, respectively. The quantitative analysis shows neuroprotective effect of naringin against KA-induced excitotoxicity in the hippocampus (b). *p* < 0.001 and *p* = 0.006, significantly different from contralateral controls and KA alone, respectively (one-way ANOVA and Tukey* post hoc* test, *n* = 4 for each group). CON: contralateral controls side; EXP: experimental side; KA: KA-treated hippocampus; NAR(80): 80 mg/kg naringin-treated hippocampus; NAR(80)+KA: 80 mg/kg naringin with KA-treated hippocampus.

We next examined whether naringin protected hippocampal neurons from KA-induced excitotoxicity. Seven days after KA treatment with daily intraperitoneal injection of naringin, mice brains were removed and sections were labeled with NeuN antibody to show neurons in the hippocampus. Our results showed that neuronal cell death was apparently observed in the CA1 area of hippocampus 7 days after KA treatment compared with contralateral controls (Figure 2(a)), which showed no difference compared with PBS-treated hippocampus (data not shown). However, treatment with naringin attenuated the loss of hippocampal neurons in the KA-treated CA1 region (Figure 2(a)). When quantified and expressed as a percentage of the CA1 neurons in the counting area of the ipsilateral hippocampus relative to the contralateral control, only 61% of the hippocampal neurons were preserved in the hippocampal CA1 region of the KA-treated rats (Figure 2(b), *p* < 0.001 versus contralateral controls), whereas 80% of the hippocampal neurons were preserved in the naringin plus KA-treated group (Figure 2(b), *p* = 0.006 versus KA alone). Naringin alone did not affect the number of hippocampal neurons compared with intact controls (data not shown). These results show that naringin protects hippocampal neurons from KA excitotoxicity.

### 3.3. Treatment with Naringin Induces a Decrease in Autophagic Stress in the KA-Treated Hippocampus

**Figure 3:**
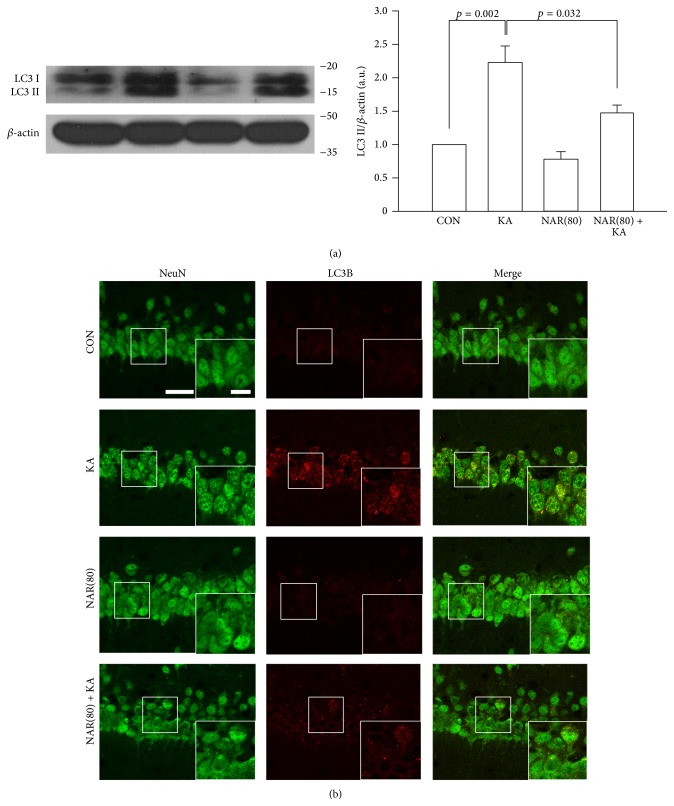
Reduction of autophagic stress by naringin treatment in the KA-treated hippocampus. Mice received twice intraperitoneal injection of naringin at 1 day and 1 h before KA treatment, and mice were sacrificed 1 day after KA treatment. Western blot analysis shows the bands of LC3 (a). There is no significant difference to the level of LC3 II by NAR(80) compared to CON. However, NAR(80) significantly reduced the level of KA-induced LC3 II. Quantitative data are calculated from the density of LC3 II bands normalized to the density of *β*-actin bands for each sample. *p* = 0.002 and *p* = 0.032, significantly different from CON and KA alone, respectively (one-way ANOVA and Tukey* post hoc* test, *n* = 4 for each group). Representative pictures of double immunofluorescence staining show LC3B-positive punctate signals within NeuN-stained neurons in the KA-treated hippocampal CA1 area (b). Naringin treatment attenuates LC3B punctate signals in the hippocampal CA1 neurons treated by KA. Scale bar: 20 *μ*m and 10 *μ*m, respectively.

To investigate how naringin attenuated the KA-induced neurotoxicity, we next examined whether treatment with naringin contributed to reduction of KA-induced autophagic stress, resulting in neuronal damage [11]. Similar to the experiments of seizure onset, mice received an intraperitoneal injection of naringin starting 1 day before KA treatment and again 1 h prior to seizure induction, and then the brain tissues were prepared for Western blot and immunohistochemical analysis of the levels of LC3 as a marker of autophagic stress at 1 day after KA treatment. Western blot analysis showed that the levels of LC3 II, which indicates induction of autophagic stress, were increased in mice treated with KA compared to intact controls (Figure 3(a), *p* = 0.002 versus CON), but the increased levels were decreased by naringin treatment (*p* = 0.032 versus KA alone). Consistent with Western blotting, immunofluorescence results showed that KA treatment induced an increase in LC3B expression in hippocampal CA1 neurons, and its overexpression was apparently decreased by naringin treatment (Figure 3(b)), suggesting that naringin might have a property of reducing autophagic stress, which could be involved in neuronal cell death.

### 3.4. Treatment with Naringin Inhibits Microglial Activation in the KA-Treated Hippocampus

**Figure 4:**
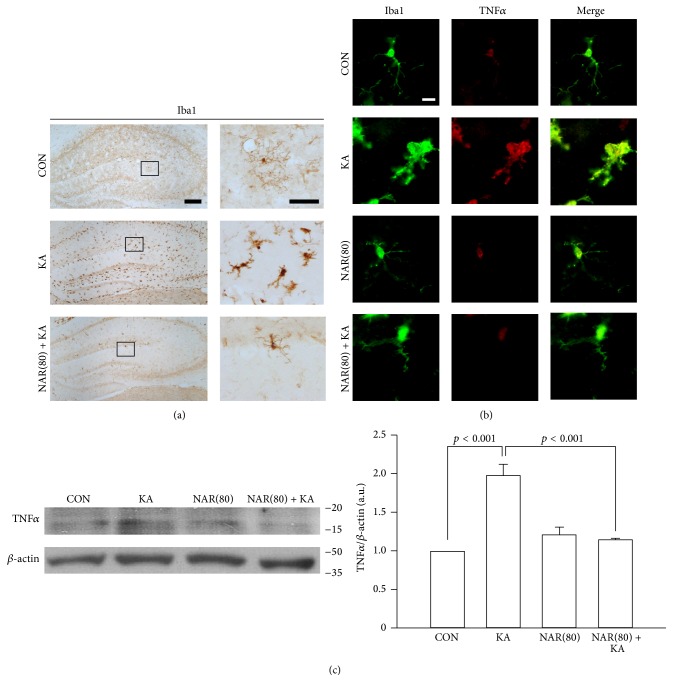
Anti-inflammatory effects of naringin in the KA-treated hippocampus. Similar to experiments for autophagic stress, mice received treatment with naringin. Representative pictures of immunoperoxidase staining for Iba1 in the hippocampus (a). Morphologic changes of KA-induced microglia activation are found at 1 day after KA treatment in the hippocampus compared with CON; however, treatment with naringin decreases microglia activation triggered by KA excitotoxicity. Scale bar: 200 *μ*m and 50 *μ*m, respectively. Consistent with naringin treatment-induced deactivation of microglia, treatment with naringin shows reduction of TNF*α* expression in Iba1-positive microglial cells of the hippocampus (b). Scale bar: 10 *μ*m. Western blot analysis shows that treatment with naringin significantly reduces the KA-increased levels of TNF*α* compared to KA alone (c). Quantitative data are calculated from the density of TNF*α* bands normalized to the density of *β*-actin bands for each sample. *p* < 0.001, significantly different from CON and KA alone, respectively (one-way ANOVA and Tukey* post hoc* test, *n* = 4 for each group).

Recent studies showed that excessive neuroinflammation may play an important role in the progression of epilepsy [3, 5]. In the previous studies, many results showed that naringin could protect neurons through anti-inflammatory and antioxidative effects in neurodegenerative diseases such as Huntington's and Alzheimer's diseases [22, 23]. Moreover, our previous study showed anti-inflammatory effects of naringin, consequently resulting in neuroprotection in the animal model of Parkinson's disease [16], suggesting its property as an inhibitor of glial activation in the brain. Thus, we further examined whether treatment with naringin contributed to inhibition of microglial activation in the KA-treated hippocampus. The results of immunohistochemistry for Iba1 showed that KA excitotoxicity activated microglial cells in the hippocampus compared to intact controls, and microglia activation following KA treatment was reduced by treatment with naringin (Figure 4(a)). In addition to morphological changes of microglia, our observations showed that naringin treatment attenuated an increase in TNF*α* within Iba1-positive microglia in the KA-treated hippocampus (Figure 4(b)). Consistent with the immunostaining results, Western blot analysis showed that treatment with naringin significantly reduced the levels of TNF*α* increased by KA treatment (*p* < 0.001 versus KA alone). Therefore, our results suggest that naringin may protect the hippocampal CA1 neurons via suppression of autophagic stress and microglial activation in the KA-treated hippocampus.

## 4. Discussion

A number of studies have shown that naringin, one of effective flavonoids, possesses protective effects through their beneficial properties such as antioxidation and anti-inflammation against neurological injuries [15, 22, 23]. We also reported that its treatment protected the nigrostriatal dopaminergic projection against neurotoxicity involved in Parkinson's disease [16]. Moreover, there is evidence showing that naringin can delay the onset of seizures induced by KA treatment, even though it is unclear whether naringin could affect development of chronic epileptic seizures [17]. In the present study, our results showed that naringin treatment significantly reduced seizure susceptibility. Furthermore, naringin significantly inhibited occurrence of SRS in KA-treated mice (Figure 1), suggesting that treatment with naringin might be useful to induce functional beneficial effects against epileptic process* in vivo*.

As described above, naringin had potential properties for antiepileptic effects against KA-induced seizures; however, it was unclear how naringin could affect KA-induced pathophysiological features such as neuronal cell death and glial activation, leading to consequent development of epileptic seizures [1, 2, 5, 24]. Recently, researchers suggest that autophagic stress induces neuronal death in* in vitro* and* in vivo* models of neurodegeneration [7, 9, 10]. Moreover, Shacka et al. suggest that activation of autophagic stress induced by KA treatment may be involved in excitotoxic cell death in the mouse hippocampus [11]. These evidences suggest that the control of KA-induced autophagic stress may be important to protect the hippocampal neurons. In the present study, our results showed that treatment with naringin protected the hippocampal CA1 neurons from KA-induced neuronal cell death (Figure 2). Furthermore, we found that naringin significantly reduced expression of LC3 in the KA-treated hippocampal neurons (Figure 3), suggesting that naringin might contribute to neuroprotection via suppression of KA-induced autophagic stress in the hippocampal neurons.

There are many reports showing that excessive expression of inflammatory cytokines released from glial cells may contribute to the progression of epilepsy [3, 5, 25]; for instance, TNF*α* released from KA-activated microglia increases excitotoxicity in the hippocampal neurons [6], suggesting that inflammatory processes may be associated with the etiopathogenesis of seizures and the cause of hippocampal cell death [26]. In the present study, our results showed that treatment with naringin attenuated microglial activation following KA excitotoxicity, resulting in a reduction of the expression of TNF*α* in the KA-treated hippocampus (Figure 4), which might be involved in protection of neurons from KA-induced excitotoxicity. Taken together, our observations suggest that treatment with naringin may have beneficial properties to protect neurons from KA-induced excitotoxic cell death viaprevention of the KA-induced pathophysiological events, such as autophagic stress and activated microglia-derived neuroinflammation in the hippocampus* in vivo*, which may at least in part be involved in the blockade of epileptogenesis.

## 5. Conclusions

In conclusion, the purpose of the present study was to investigate the beneficial properties of naringin for antiexcitotoxicity in the KA-induced mice model of epilepsy. Our results showed that treatment with naringin could significantly decrease seizure activities in KA-treated mice and attenuated neuronal cell death, autophagic stress, and microglial activation in the KA-treated hippocampus. Although further studies are necessary to demonstrate beneficial effects by naringin treatment in the chronic epileptic hippocampus, our observations suggest that naringin may be a beneficial natural product for treatment of epilepsy.
